# Round the Hospitals

**Published:** 1916-02-05

**Authors:** 


### Round the Hospitals.

Public interest in nurses and nursing has been raised to a point higher than has hitherto been reached owing to the award by the King last month of eighty-five Royal Red Cross decorations to members of the Nursing Services representing almost every department of the work. The honoured recipients included members of Queen Alexandra's Imperial Military Nursing Service and the Reserve, the Civil Hospital (Reserve), the Territorial Force Nursing Service, the British Red Cross, the Service for India, and the Australian, New Zealand, and Canadian Nursing Services. The war has stimulated interest and awakened energies and efforts on the part of individual nurses of all ranks, from the humblest to the highest, which have resulted in an immensity of good not only to individual workers and their patients, but to everyone who has had the good fortune to be brought into touch with the atmosphere thus created.

Our readers are by this time familiar with the names of the recipients as recorded in the Honours *Gazettes*, and they have probably read biographical notices of individual principal matrons, matrons, sisters, and nurses. The attention thus directed to the Nursing Services has a special value. It not only stimulates endeavour, but it emphasises the value of self-denying effort and great administrative gifts. But there is another side to the work done by women since the war as attendants upon the sick. The demands made upon individual workers have never been so great, except perhaps occasionally in times of sudden and extensive epidemics of the severest type. Even then the strain has usually been limited to a few weeks, or, once and again, to months; but the war has lasted already exactly a year and a half.

Again, the work that the nursing profession have had to undertake since the war has had to be done under an infinite variety of circumstances. It has often entailed a necessity for the worker to keep going continuously through long hours, frequently in excess of any permitted by the time-tables which have governed nurses' hours of labour in times of peace. Wherever wounded patients have been received, there the call upon the nurses can seldom be a fixed quantity, for the hour of arrival of batches of wounded is necessarily often after midnight, in the early morning, and even on Christmas Day or other general holidays. We know of one case of a voluntary hospital where the matron and staff had just settled their patients and nurses in one of the large wards which had been converted into a theatre for the occasion. The performance had scarcely commenced when a large batch of wounded men arrived from the Front, without any previous notice, and they had to be taken in and accommodated in the absence of any special provision for their reception and accommodation. Yet no sign of irritation or upset was exhibited by a single member of the staff, medical or nursing. With the utmost order, in the minimum of time, the difficulties were faced and solved, with a cheerfulness and zeal which were characteristic of the spirit which has everywhere prevailed throughout our hospitals in this country during war-time.

The responsibility and actual burden of work which have devolved upon the matrons and sisters of the voluntary hospitals admitting wounded warriors cannot be exaggerated. Many of the matrons of our great voluntary hospitals up and down the country have not only faced the burden of additional duties due to the provision of extra beds and large increases in the number of patients under treatment, but they have cheerfully undertaken the honorary duties of principal matron to some large Territorial hospital, and have faced the strain of finding and selecting the matron, sisters, and nursing staff for it. How splendidly these principal matrons have risen to the occasion the *Gazettes* testify by recording that seventeen principal matrons have been awarded the Royal Red Cross.

It is one thing to have been brought up with and to have grown accustomed to the administration of a great voluntary hospital under ordinary conditions. It is quite another to find oneself responsible for the nursing department of the same institution when its beds have been increased by hundreds, and the administration has been modified by the introduction of military discipline and system into a portion, at any rate, of the establishment under the matron's control. It speaks volumes for the high standard of personal conduct and character attained by the matrons of our hospitals in this country that, on the whole, they have risen splendidly to the occasion, faced all the difficulties, overcome all the dangers, and won a signal triumph by producing everywhere a condition of efficiency, so high, as to make it as remarkable as it is satisfactory.

It may be well to indicate the variety of conditions which prevail at the present time in hospitals to which the wounded are admitted. In the voluntary hospitals where a certain number of beds have been provided, or others have been specially set up, for the reception of the wounded, the matron may find herself in charge of a much larger establishment, but may also be free from military discipline and the mixed system of administration so involved. Such a matron, being in charge of a large voluntary hospital, may, of course, have accepted service as a principal matron to a Territorial or other hospital, which would give her an insight into military methods and requirements, but she would be free then from the difficulties just indicated, where the two systems are at work in the same voluntary hospital. We have had some eloquent testimony of the great honour due to the sisters in many a voluntary hospital who have remained at their posts, often under very trying circumstances, though tempted to take work directly connected with the War Office, which, if not more interesting from a nursing point of view, is regarded by many as more important, because it is war work, if you please, as the saying is, and would receive due recognition—that is, a decoration or a medal. We may here ask this question: If nursing work done directly under the War Office, or through the Red Cross, or in a Territorial military hospital, is war work, what is work in the wards of a voluntary hospital, or the temporary buildings attached to it, which are devoted entirely to the treatment of sailors or soldiers? Having regard to the quality of this latter work, and the courage and conscience exhibited by sisters of the type just referred to, is it not time that someone in authority should spontaneously provide that all sisters during war-time working in wards occupied by wounded sailors or soldiers shall have the privilege of wearing a ribbon or collar-strap or some distinctive mark that they, too, are on war service, the same as those working in Military, Territorial, Red Cross, and other establishments more directly connected with the Admiralty or the War Office?

### Miss C. A. Tait McKay, R.R.C.

We sincerely regret that in the article "Patriotism and the Nurse Training Schools," published in our issue of January 22, page 375, although Miss Tait McKay's name was included among the list of nurses trained at Guy's Hospital attached to military hospitals, it was accidentally omitted from the distinguished matrons on whom the Royal Red Cross has recently been conferred by the King. Miss McKay is the matron of the 4th Southern General Hospital, at Plymouth, which has proved itself to be one of the most actively administered, efficient, and knowledgable of military hospitals. Although seventeen principal matrons of Territorial hospitals received the Royal Red Cross, only six matrons attached to Territorial hospitals were so honoured in the recent *Gazettes*. This fact demonstrates conclusively the high reputation Miss McKay has earned for herself as matron.

